# Reversibility of Sinus Bradycardia-Induced Syncope Resulting From Low-Voltage Electrical Injury: A Case Report

**DOI:** 10.7759/cureus.50509

**Published:** 2023-12-14

**Authors:** Luai Alhazmi

**Affiliations:** 1 Department of Medicine, Faculty of Medicine, Jazan University, Jazan, SAU

**Keywords:** cardiac monitoring, arrhythmia., syncope, sinus bradycardia, electrical injury

## Abstract

Electric shocks pose a serious threat to public health. The heart is among the organs that are most commonly impacted. Electrical harm can cause a number of potentially fatal heart conditions, including asystole, ventricular fibrillation, and myocardial rupture. Some patients had sinus bradycardia diagnosed at the time of admission*. *In this case report, we describe a 43-year-old male patient who had an electrical injury that resulted in syncope and sinus bradycardia. After 24 hours of cardiac monitoring, the patient was found to not require a pacemaker. This suggests that patients with symptomatic sinus bradycardia should have cardiac monitoring. If, after 24 hours, cardiac monitoring revealed no new episodes of sinus bradycardia and the patient remained asymptomatic, the patient is unlikely to require a pacemaker. There are differing guidelines and suggestions regarding the supervision of patients following electrical damage, and further study in this area is necessary to enable the unification of guidelines.

## Introduction

Electrical injuries vary greatly in severity based on a variety of factors. From mild skin burns to potentially fatal damage to key organs, electrical injuries can cause a wide variety of harm. The vast majority of available epidemiological data concerns incidents that occur while individuals are working [1]. Electrically injured patients are thought to have a higher chance of developing cardiac arrhythmias. Arrhythmias that occur due to the proarrhythmic effects of electric shock typically manifest promptly following the onset of the incident [2]. Sinus bradycardia after electrical injury has been reported previously; however, most of them are asymptomatic [3]. This report describes a patient who experienced electrical damage that resulted in sinus bradycardia and syncope, along with management strategies.

## Case presentation

**Figure 1:**
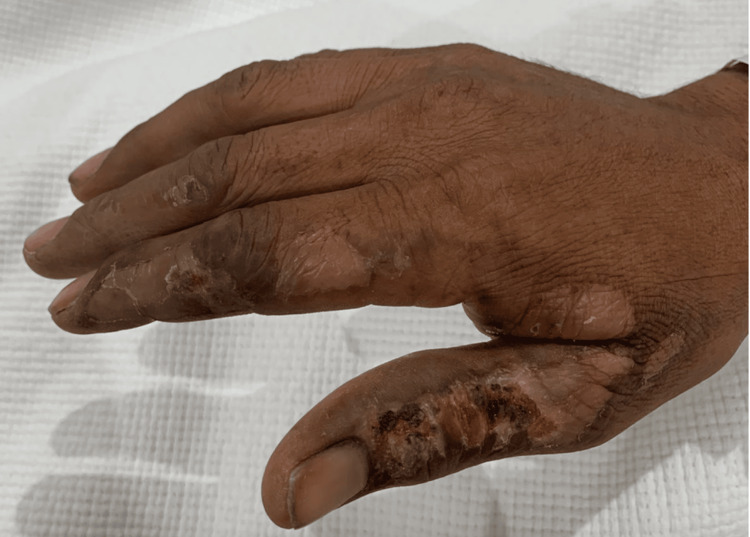
Right hand with burn injuries after electrical shock

A 43-year-old man with no medical history presented to the emergency department (ED) after an episode of syncope following an electrical injury. He had touched an electrical socket carrying 220 volts during its repair. He had lost consciousness for a few seconds after the accident but was conscious on admission. He denies any chest pain or shortness of breath; there was no palpitation. Upon physical examination, he showed no orthostatic changes, a heart rate of 42 beats per minute, and a blood pressure of 100/70 mmHg. His right hand had a burn injury (Figure 1). Auscultation showed no additional sounds and normal lung and heart sounds.

**Figure 2:**
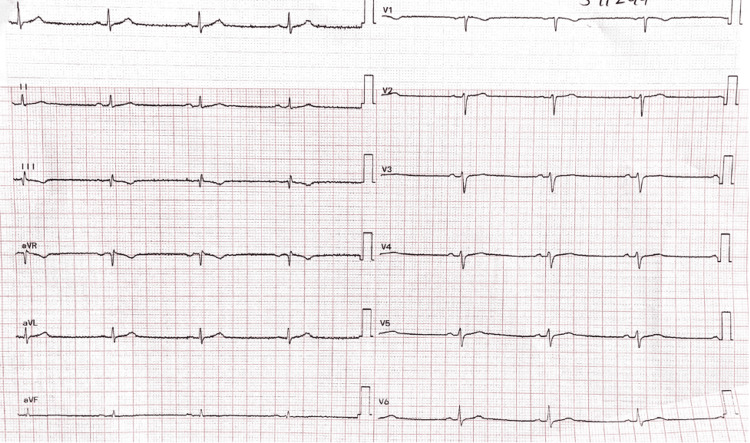
Surface 12-lead ECG taken while the patient was at rest

An ECG revealed 42 beats per minute of sinus bradycardia (Figure 2). The chest X-ray showed nothing unusual. All laboratory workup parameters, such as creatine kinase-MB, thyroid-stimulating hormone (TSH), T3/T4, calcium, magnesium, and serum potassium, were within normal ranges.

For the purpose of electrocardiographic monitoring, the patient was admitted to the coronary care unit (CCU). With the lowest rate of 40 beats per minute, sinus bradycardia was the most prevalent rhythm on telemetry. Following an echocardiography, no structural abnormalities were observed, and the left ventricular ejection fraction (LVEF) remained unchanged. The patient was monitored in the CCU for a few days with no further episodes of syncope. On the second day, his heart rate was 62 beats per minute. The patient was discharged and followed up for one year with no further episodes of syncope or any cardiac symptoms.

## Discussion

Arrhythmias caused by electrical currents are mysterious in nature. The heart is especially vulnerable to damage from electrical current because of the way it preferentially flows down blood vessels and neurons due to differences in electrical resistance. Injuries to the heart can occur from both horizontal (hand-to-hand) and vertical (hand-to-foot or head-to-foot) current passages [4].

The most prevalent cardiac consequence of electric injury is arrhythmia. Although delayed ventricular arrhythmias have also been documented, the majority of arrhythmias happen shortly after the electrical shock. While ventricular tachycardia and atrial fibrillation have also been documented, sinus tachycardia and premature ventricular contractions are much more frequent [5]. 

In a study that involved 480 patients, sinus bradycardia was identified in approximately 10.4% of patients at the time of admission. On the other hand, none of them displayed any symptoms. One hundred and eight two patients (37.9%) underwent ECG monitoring in the ED. No clinically significant arrhythmias were seen during the ECG monitoring, with the exception of two cases: one with paroxysmal atrial fibrillation and one with regular supraventricular tachycardia that was stopped by a vagal technique. Mortality within 30 days and in the hospital was 0% [3].

Regarding cardiac monitoring in the intensive care unit versus discharge from the emergency department, in 2017, the European Society of Cardiology came up with specific criteria for apparently healthy patient management after an electric accident that needed to be monitored [6]. A patient who presents with a loss of consciousness is considered high-risk and needs monitoring for 24 hours. Our patient had burn injuries from a low-voltage electrical shock; nevertheless, he was unconscious for some time after the incident. The patient was admitted to the CCU for cardiac monitoring; no arrhythmias were recorded after 24 hours of cardiac monitoring. The next day, he was completely asymptomatic, with a heart rate of around 62 beats per minute. The patient was discharged and followed up for one year with no further episodes of syncope or any cardiac symptoms.

## Conclusions

Electrical injuries can cause a wide variety of arrhythmias, including sinus bradycardia, among other severe cardiac dysfunctions. Most sinus bradycardia cases are asymptomatic and self-limiting. To the best of our knowledge, this is the first case of sinus bradycardia causing syncope related to an electrical injury to be published. This is the examination of the possibility of reversibility of bradycardia and improvement without the need for a pacemaker.* *This suggests that cardiac monitoring is needed for patients who come in with symptomatic sinus bradycardia, and if 24 hours of cardiac monitoring show no more bouts of sinus bradycardia and the patient remains asymptomatic, it is unlikely that the patient will need a pacemaker. More study is needed to unify the guidelines and recommendations for patient observation after electrical damage.
